# Texas State Medical Association

**Published:** 1902-01

**Authors:** 


### Texas State Medical Association.

The following circular has been sent to all the members of the Texas State Medical Association:

OFFICE OF PRESIDENT TEXAS STATE
MEDICAL ASSOCIATION.

BELTON, TEXAS, December 21, 1901.

*Dear Doctor:*

Owing to the unusual conditions arising with reference to our laws governing the T. S. M. A., and changes that will be brought up at our next meeting in April, to make the same conform to those of the A. M. A., and also the report of the Committee on Revision of our organic laws, it is desirable and necessary that we should have the fullest attendance possible at our next meeting. It seems that a large number of the members and many of the district and county associations believe that such an attendance could not be had at El Paso, giving their reasons as follows: El Paso is located on the extreme boundary line of Texas and in a section of country where the number of physicians is comparatively limited. The time and expense to the majority of our members would at this particular juncture, owing to the scarcity of money—the result of bad crops—almost insure a very light attendance at El Paso. These reasons I give as those submitted for consideration by several different medical societies and many members of the T. S. M. A.

The matter having been brought to the attention of the medical profession of El Paso, the following resolutions were adopted by their county society:

"AT THE LAST REGULAR MEETING OF THE EL PASO COUNTY MEDICAL SOCIETY OF EL PASO, TEXAS, THE FOLLOWING RESOLUTION WERE ADOPTED:

"December 14, 1901.

"WHEREAS, The El Paso County Medical Society has been advised by the officers and other members of the T. S. M. A. that in their opinion it would be detrimental to the interests of the Association to hold the annual meeting for 1902 in El Paso, for the following reasons:

"First. The next meeting of the T. S. M. A. will be one of more than ordinary importance, and should have as large an attendance as possible on account of changing the laws of the Association to conform to those of the A. M. A.

"Second. Owing to the almost complete failure of all crops over the entire State this year (1901), a great majority of the members of the Association cannot afford the expense of the trip to El Paso.

*"Now, be it resolved* by the El Paso County Medical Society, for the reasons above stated, it is willing to forego the honor and pleasure of having the 1902 meeting of the T. S. M. A., if the officers and fathers of the Association are convinced that the interests of the Association can be better subserved by holding this meeting at a more central point in the State. We are willing to leave the matter entirely with them, feeling that they will be fair to us and just to the Association.

"S. T. TURNER, President.

"J. A. RAWLINGS, Secretary."

This spirit of unselfishness and loyalty on the part of the El

Paso fraternity is in keeping with the reputation that the people and physicians of that city have always enjoyed of being broad, liberal and patriotic in all their undertakings.

I enclose you a self-addressed postal card. Please give us your vote on the following points: Shall we change the place of meeting? If so, where shall we meet?

It is the desire of your officers to get a full concensus of opinion and determine what is to the best interest of the Association in settling this question. Let me hear from you as soon as possible.

Very respectfully,

Taylor Hudson, M. D.,

President T. S. M. A.

